# Determination of technology-critical elements in seafood reference materials by inductively coupled plasma-tandem mass spectrometry

**DOI:** 10.1007/s00216-023-05081-z

**Published:** 2023-12-23

**Authors:** Dominik Wippermann, Alexa Zonderman, Tristan Zimmermann, Daniel Pröfrock

**Affiliations:** 1 Department Inorganic Environmental Chemistry, Helmholtz-Zentrum Hereon, Institute of Coastal Environmental Chemistry, Max-Planck-Str. 1, 21502 Geesthacht, Germany; 2 Department of Chemistry, Inorganic and Applied Chemistry, Universität Hamburg, Martin-Luther-King-Platz 6, 20146 Hamburg, Germany; 3 Department of Biology, Marine Ecosystem and Fishery Science, Universität Hamburg, Olbersweg 24, 22767 Hamburg, Germany

**Keywords:** ICP-MS/MS, Marine reference materials, Microwave digestion, Rare earth elements, N_2_O collision/reaction cell

## Abstract

The certified reference materials (CRMs) BCR-668 (mussel tissue), NCS ZC73034 (prawn), NIST SRM 1566a (oyster tissue) and NIST SRM 2976 (mussel tissue) were analyzed for their mass fractions of 23 elements using inductively coupled plasma tandem-mass spectrometry (ICP-MS/MS). This study focused on the quantification of selected technology-critical elements (TCEs), specifically rare earth elements (REE) and the less studied TCEs Ga, Ge, Nb, In and Ta. Microwave assisted closed vessel digestion using an acid mixture of HNO_3_, HCl and H_2_O_2_ was applied to varying sample masses and two different microwave systems. Recoveries of 76% (Gd, NCS ZC73034) to 129% (Lu, BCR-668) were obtained for the REE and 83% (Ge, NCS ZC73034) to 127% (Nb, NCS ZC73034) for the less studied TCEs across all analyzed CRMs (compared to certified values) using the best-performing parameters. Mass fractions for all analyzed, non-certified elements are suggested and given with a combined uncertainty *U* (*k* = 2), including mass fractions for Ga (11 µg kg^-1^ ± 9 µg kg^-1^ to 67 µg kg^-1^ ± 8 µg kg^-1^) and In (0.4 µg kg^-1^ ± 0.3 µg kg^-1^ to 0.8 µg kg^-1^ ± 0.7 µg kg^-1^). This study provides mass fractions of possible new emerging contaminants and addresses the relevant challenges in quantification of less studied TCEs, thus allowing the application of existing CRMs for method validation in studies dealing with the determination of TCEs in seafood or other biota.

**Graphical abstract:**

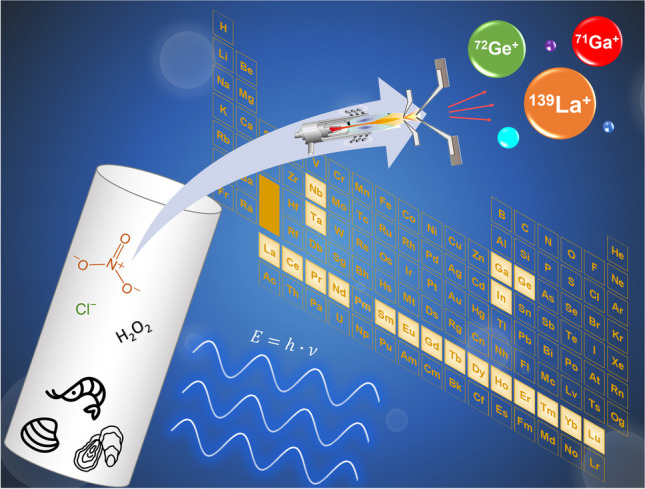

**Supplementary Information:**

The online version contains supplementary material available at 10.1007/s00216-023-05081-z.

## Introduction

Technology-critical elements (TCEs), such as Ga, Ge, Nb, In, Ta and rare earth elements (REE) have undergone a dramatic increase in industrial applications in recent years. The electronics industry in particular has been subject to considerable change in terms of the demand for raw materials, from approximately a dozen elements in regular use in the 1980s to almost every element of the periodic table in current technologies [1]. Even though classical environmental pollutants like Pb or Zn show higher annual rates of production (e.g. in 2018 Pb: 4.3 10^6^ t a^-1^ vs. ∑REE: 2.19 10^5^ t a^-1^), the increasing global application of TCEs may lead them to become contaminants of concern in the near future [2]. This situation is exacerbated by the poor or nonexistent recycling rates of many TCEs, and as a result annual global TCE waste rises similarly to production [3].

Resulting in TCE waste input into the marine environment either directly or indirectly via anthropogenic activities at sea or transport of industrial pollutants. A current example of TCE use in the marine environment is the construction of offshore wind turbines and the related usage of corrosion protection systems. For example, the TCEs Ga and In are used in alloying components of sacrificial anodes, and their implementation among other considerations has led to the investigation of the possible environmental impacts of corrosion protection on the marine environment [4, 5]. While current literature does not indicate significant inputs from this source [6, 7], the increasing demand for renewable energy is expected to drive demand and outputs [8], justifying interest in TCE waste generated by offshore activities, leading to the investigation of possible environmental impacts [7, 9, 10]. Indirectly, rivers can act as input sources of TCEs from nearby industry. Von der Au *et al.* found elevated Ga mass fractions in German North Sea surface sediments in 2014 when compared to historical data from 1980, confirming riverine transport of TCEs [11].

To be able to monitor anthropogenic emissions of TCEs into the environment, reliable and accurate analytical measurement procedures are necessary. However, the determination of TCE mass fractions can be complex and challenging. This is due to the low abundance of TCEs in many environmental matrices (the abundance of In in the upper continental crust is only 56 ng g^-1^ [12]), and the complex chemical and mineralogical composition of environmental matrices. Specifically, in inductively coupled plasma mass spectrometry (ICP-MS) analysis matrix components can cause isobaric, polyatomic and/or doubly charged interferences. In fact, not only other elements, but also other TCEs may cause interferences, for example ^142^Ce^2+^, ^141^Pr^2+^, and ^142^Nd^2+^ which act as interferences for ^71^Ga^+^. Therefore, it is not surprising that many of the methods found in current literature describe only single or few selected TCEs as target analytes [13–15]. However, advances in the use of inductively coupled plasma tandem-mass spectrometry (ICP-MS/MS) together with a reaction gas can eliminate the majority of these interferences [16]. Recent studies have demonstrated the advantages of N_2_O as a reaction gas over conventionally used O_2_ for the development of a multi-element method which covers all major TCEs [17, 18]. Lancaster *et al*. showed that 59 elements achieved improved sensitivity when performing mass-shift with N_2_O rather than O_2_. Additionally, N_2_O demonstrated a collisional focusing effect for 36 elements when measuring on-mass, whereas this was not the case for O_2_ [19].

Even though recent studies engage these challenges, another serious problem in evaluating environmental data is the lack of reliable reference values and background data. Numerous legal directives offer maximum values for common pollutants (e.g. Marine Strategy Framework Directive 2008/56/EG and Water Framework Directive 2000/60/EC), however at the current time only classical pollutants such as Ni, Cu, Zn, Cd, Hg, Pb are regulated [20, 21]. Similar trends can be observed in global agreements for a clean environment, including the Oslo-Paris Agreement (OSPAR), which does not explicitly consider possible emerging contaminants at the moment [22]. Furthermore, many available certified reference materials (CRMs) do not include mass fractions of TCEs in their certificate, making it difficult to validate measurement procedures. To the best of our knowledge, only three articles which propose mass fractions for noncertified TCEs within the presented CRMs have been published up to this point (July 2023). An overview of the available literature for the selected commonly used seafood CRMs (crustaceans, mollusks and fish) can be found in the Supplementary Information 1 (Table ESM 1). Therefore, it is not surprising that in recent years few studies were published which investigated TCE mass fractions in marine organisms (especially, the less studied TCEs Ga, Ge, Nb, In, Te or Ta), further complicating the assessment of their impact and significance on the marine environment [23].

This study provides mass fractions of possible emerging contaminants and addresses the analytical challenges presented by TCEs for ICP-MS/MS analysis. The method is evaluated using four seafood reference materials: BCR-668 (mussel tissue), NCS ZC73034 (prawn), NIST SRM 1566a (oyster tissue) and NIST SRM 2976 (mussel tissue), from which two have certified values for all REEs (BCR-668 and NCS ZC73034) and one for the less studied TCEs Ge and Nb (NCS ZC73034). Parameters offering best results are subsequently applied to suggest mass fractions of 19 TCEs within this set of CRMs. These suggested values facilitate the application of existing CRMs for method validation in future studies dealing with the measurement of TCE mass fractions in seafood and other biota.

## Material and methods

### Reagents and standards

Specific preparation steps of the laboratory work were performed within a class 1000 clean bench. Type I reagent grade water (>18.2 MΩ cm) was obtained from an ultrapure water system consisting of an Elix 3 module (Merck Millipore, Darmstadt, Germany), a Milli-Q Element module (Merck Millipore, Darmstadt, Germany) and a Q-POD element (Merck Millipore, Darmstadt, Germany). HNO_3_ (65% *w*/*w*, Carl Roth GmbH & Co. KG, Karlsruhe, Germany) and HCl (37% *w*/*w*, Carl Roth GmbH & Co. KG, Karlsruhe, Germany) were further purified by double sub-boiling in perfluoralkoxypolymer (PFA)–stills (DST 4000 & DST 1000, Savillex, Minnesota, USA). Ultra-pure H_2_O_2_ (31% w/w, Carl Roth GmbH & Co. KG, Karlsruhe, Germany) was used without further preparation. Quantification was performed via external calibration, which was prepared from single element standards for Ge, Nb, In, Ta (Merck, Darmstadt, Germany) and custom-made multi-element calibration standards of varying composition for Ga and REE (Inorganic Ventures, Christiansburg, USA). Certificates of used standard solutions are traceable to NIST SRMs.

The reference material BCR-668 (mussel tissue) (Institute for Reference Materials and Measurements, Geel, Belgium, the reference material NCS ZC73034 (prawn) (National Analysis Center for Iron and Steel, Beijing, China) and the reference materials NIST SRM 2976 (mussel tissue) and NIST SRM 1566a (oyster tissue) (National Institue of Standards and Technology, Gaithersburg, USA) were measured for validation purposes.

### Moisture content of reference materials

Reference materials were dried according to the instructions given in the certificate. Separate subsamples were used to determine the residual moisture. For BCR-668 one subsample of 1 g was used according to the certificate. For the remaining reference materials triplicates of the minimum sample portion were used. Obtained moisture content was subtracted from the weighed sample. Further information concerning drying technique and the respective moisture content of each analyzed reference material can be found in Table 1.

**Table 1:** Overview of the moisture content of BCR-668, NCS ZC73034, NIST SRM 1566a and NIST SRM 2976 obtained according to the drying instructions given in the certificate

Reference material	Drying technique (adapted from certificate)	Test portion size / g	Moisture content / %
BCR-668	Oven at 80 °C until constant mass	1.0 (*n*=1)	2.2
NCS ZC73034	Oven at 80 °C for 4 h	0.20 (*n*=3)	9.9 ± 0.5
NIST SRM 1566a	Freeze drying for 20 h at 1 Pa	0.25 (*n*=3)	6.34 ± 0.18
NIST SRM 2976	Oven at 80 °C until constant mass	0.5 (*n*=3)	9.1 ± 0.3

### Sample digestion

Samples of 100 mg were digested using a MARS 6 microwave system (CEM Corp., Kamp Lintfort, Germany) in 55 mL acid vapor cleaned TFM vessels. Samples were digested using 5 mL HNO_3_, 2 mL HCl and 1 mL H_2_O_2_ at 200 °C for 300 min according to a microwave program adapted from Zimmermann et al. [24]. Samples of 100 mg, 250 mg and 500 mg were digested using a Multiwave 7000 microwave system (MW 7000, Anton Paar, Ostfildern, Germany) in conjunction with 18 mL acid vapor cleaned quartz vials. Samples of 100 mg were digested with 5 mL HNO_3_ and 2 mL HCl according to the microwave program presented in Table 2. Samples of 250 mg and 500 mg were digested using an additional digestion step: following the first digestion, an additional 2 mL of HNO_3_ was added to each quartz vial followed by the repetition of the digestion program. After digestion, samples were quantitatively transferred to 50 mL graduated polypropylene vessels (DigiTUBE^®^; SCP Science, Quebec, Canada) precleaned with HNO_3_ (2% *w*/*w*) and filled to a total volume of 50 mL with type I reagent grade water. TFM vessels were rinsed three times with type I reagent grade water followed by an acid vapor cleaning using an ETC EVO II (ANALAB, Hoenheim, France) with HNO_3_ (65% *w*/*w*) and type I reagent grade water. Quartz vials were cleaned by running a blank digestion using the same acid mixture, rinsing three times with type I reagent grade water, and subsequently acid vapor cleaning using an ETC EVO II (ANALAB, Hoenheim, France).

**Table 2:** Microwave programs used for sample dissolution. Parameters for MARS 6 method are based on [24]

Multiwave 7000				
5 mL HNO_3_, 2 mL HCl (+ additional 2 mL HNO_3_ for the 250 mg and 500 mg sample mass)				
Temperature / °C	Pressure / bar	Ramp. / min	Hold / min	Sample weight / mg
280	140	15	60	100; 500 (250 for BCR-668)
MARS 6				
5 mL HNO_3_, 2 mL HCl, 1 mL H_2_O_2_				
Temperature / °C	Pressure / bar	Ramp. / min	Hold / min	Sample weight / mg
200		30	300	100

### Instrumentation and measurement procedures

Multielemental analysis was conducted using an ICP-MS/MS (Agilent 8800, Agilent Technologies, Santa Barbara CA, USA) coupled to an ESI SC-4 DX FAST autosampler (Elemental Scientific, Omaha, Nebraska, USA) utilizing a loop volume of 1.5 mL. Detailed information about the operating parameters and cell gas modes of the ICP-MS/MS, as well as quantified mass-to-charge ratios and their detection modes can be found in the Supplementary Information 1 (Table ESM2 and ESM3). H_2_, He and N_2_O were employed as cell gas modes for the analyzed elements, whereby H_2_ and N_2_O acted as reaction gases in MS/MS mode. Quantified mass-to-charge ratios and corresponding cell modes were selected based on achieved sensitivity, as well as by occurrence of isobaric and polyatomic interferences. Additional information about N_2_O as a reaction gas for the determination of TCE mass fractions and potential isobaric interferences, as well as reviews about the working principles of ICP-MS/MS can be obtained elsewhere [17, 25, 26]. A tune solution containing Li, Co, Y, Ce and Tl (10 µg L^-1^) was used to regularly optimize the instrument. External calibration prepared on a daily basis was used for quantification covering a concentration range from 0.1 µg L^-1^ to 100 µg L^-1^ for each analyte. Potential carry-over effects were monitored by measuring wash blanks (2% HNO_3_ (*w*/*w*)) after each triplicate of samples.

### Data processing and calculations

Multi-element data were processed using MassHunter version 4.4 (Agilent Technologies, Santa Barbara CA, USA) and evaluated using a custom-written Excel^©^ spreadsheet. Limits of detection (LOD) and limits of quantification (LOQ) (see Table 3) were calculated from procedural blanks, including three times the standard deviation (3 x SD) for LOD and ten times (10 x SD) for LOQ according to MacDougall *et al.* [27]. Outliers were determined according to Dean and Dixon (*P* = 0.95) and were not included in further evaluation of the data [28]. Combined uncertainties (*U*, *k* = 2) were calculated for each sample replicate according to Reese *et al.* based on a simplified *Kragten* approach, taking into account repeatability precision from the instrumental measurement results and the intermediate precision obtained from the measurement results after multiple samples [29, 30]. The uncertainty for each mass fraction and significant number of digits for each value are presented according GUM and EURACHEM guidelines [31, 32]. Measurement results are compared to certified values following an approach of the European Commission (ERM) taking into account the absolute difference between mean measured value and certified value and the combined uncertainty of result and certified value [33]. If this approach is not possible (due to missing information in the certificates) certified values are discussed based on recoveries. For indicative values no recoveries were calculated. Calculations according to ERM can be found in Supplementary Information 2.

**Table 3:** LOD and LOQ for all quantified elements for the used microwave systems

Element	Anton Paar MW 7000 (quartz vials) *n*=6		CEM MARS 6 (TFM vessels) *n*=8	
	LOD [µg L^-1^]	LOQ [µg L^-1^]	LOD [µg L^-1^]	LOQ [µg L^-1^]
Mg	7	22	4	11
Al	700	2500	9	30
Zn	1.4	5	0.5	1.4
Ga	0.07	0.25	0.011	0.017
Ge	0.03	0.09	0.009	0.017
Nb	0.05	0.16	0.0010	0.0020
Cd	0.05	0.16	<0.00001	<0.00001
In	0.026	0.06	0.013	0.018
La	0.08	0.27	0.0010	0.004
Ce	0.15	0.5	0.003	0.009
Pr	0.022	0.07	0.0010	0.0020
Nd	0.05	0.18	0.0010	0.004
Sm	0.019	0.06	0.0010	0.003
Eu	0.008	0.028	0.00020	0.0010
Gd	0.017	0.06	0.0020	0.006
Tb	0.009	0.029	0.00020	0.0010
Dy	0.020	0.07	0.0010	0.0020
Ho	0.009	0.03	0.00005	0.00013
Er	0.016	0.05	0.0010	0.0020
Tm	0.009	0.029	0.00020	0.0010
Yb	0.016	0.05	<0.00001	<0.00001
Lu	0.007	0.025	0.00006	0.00017
Ta	0.009	0.029	0.0006	0.0017

## Results and discussion

### Assessment of the digestion vessels

The two primary aspects to be considered in terms of digestion vessel material are the effectiveness of cleaning strategies, therefore optimizing blank values and the inert behavior of the vessels towards relevant chemicals. For mixtures of HCl, HNO_3_ and H_2_O_2_, as used in this study, both quartz glass and TFM polymer are suitable and show sufficient durability. Differences within the blank concentrations were found, leading to LODs and LOQs at least one order of magnitude lower using the MARS 6 system together with TFM polymer vessels when compared to LODs and LOQs using the Multiwave 7000 system together with quartz vials (see Table 3).

A number of the quartz vessels provided by Anton Paar had been previously used for other demonstration purposes. Therefore, all vessels were prepared by acid vapor cleaning and a single subsequent blank digestion before usage. Blanks were randomly chosen from all provided vessels. Therefore, blanks represent a mixture of pre-used and new vessels. Overall, the cleaning resulted in sufficiently low blank values for the purpose of this study. If necessary, blanks could be improved either by regular blank digestions or a leaching step using diluted HF. We would like to note that TFM vessels for the Multiwave 7000 exist, but were not available for this study.

### Method development

Initial experiments were conducted to ensure the best results in terms of sample size and digestion procedure. Sample portions of 500 mg and 250 mg for BCR-668 were digested using the Multiwave 7000 system, exceeding the minimum recommended sample amount of all reference materials. The higher sample portion size was solely tested within the Multiwave 7000 system, since high amounts of organic material can lead to venting of the used TFM vessels by overpressure. Digestion vessels specifically designed for higher digestion pressures are available for CEM microwave system but were not available in this study. Additionally, sample portions of 100 mg were digested for each reference material in two different microwave systems with varying digestion temperatures (MARS 6 microwave system with TFM vessels and Multiwave 7000 microwave system with quartz vials), falling below the minimum recommended sample size for each reference material, except BCR-668. Thus, it is necessary to evaluate whether a smaller test portion also leads to valid results, in particular because of the limited amount of dried mussel tissue in real-life samples. To compare digestion efficiency, recoveries of six elements are discussed: Al and Mg as bulk elements, Cd and Zn as classic pollutants and Ce and Eu as representatives for rare earth elements and TCEs (see Table 4).

**Table 4:** Certified (printed in bold (*indicative/noncertified)) and measured mass fractions (dry mass) of BCR-668, NCS ZC73034, NIST SRM 1566a and NIST SRM 2976 (minimum sample weight: 0.1 g, 0.2 g, 0.25 g, 0.5 g, respectively) for Al and Mg as bulk elements, Cd and Zn as classic pollutants and Ce and Eu as rare earth elements/TCEs. Errors of the measured mass fractions correspond to the expanded uncertainty U (k = 2). # indicate measured mean values not significantly different from the certified value based on [33]

CRM and Method	Al / g kg^-1^ [Recovery / %]	Mg / g kg^-1^ [Recovery / %]	Cd / mg/ kg^-1^ [Recovery / %]	Zn / mg kg^-1^ [Recovery / %]	Ce / µg kg^-1^ [Recovery / %]	Eu / µg kg^-1^ [Recovery / %]
**BCR-668**						
**Certified** (*indicative/noncertified)	**-**	**-**	*0.275 ± 0.011	*70.7 ±0 .4	**89 ± 7**	**2.79 ± 0.16**
MARS 6 [100 mg] *n=16*	0.0196 ± 0.0022 [-]	3.9 ± 0.9 [-]	0.32 ± 0.10 [-]	68 ± 10 [-]	87 ± 21 [98]^#^	2.6 ± 1.6 [93]^#^
MW 7000 [100 mg] *n* =6	0.011 ± 0.019 [-]	3.19 ± 0.14 [-]	0.35 ± 0.07 [-]	73.5 ± 2.8 [-]	110 ± 50 [124]^#^	2.6 ± 1.8 [93]^#^
MW 7000 [250 mg] *n* =6	0.05 ± 0.07 [-]	4.43 ± 0.18 [-]	0.27 ± 0.04 [-]	56 ± 6 [-]	92 ± 23 [103]^#^	2.6 ± 0.8 [93]^#^
**NCS ZC73034**						
**Certified** (*indicative/noncertified)	*0.29	**1.69 ± 0.006**	**0.039 ± 0.002**	**76 ± 4**	**130 ± 30**	**2.5 ± 0.3**
MARS 6 [100 mg] *n*=7	0.13 ± 0.04 [-]	1.5 ± 0.3 [89]	0.037 ± 0.019 [95]	90 ± 18 [118]	126 ± 15 [97]	1.7 ± 1.2 [68]
MW 7000 [100 mg] *n* =6	0.26 ± 0.14 [-]	1.21 ± 0.06 [72]	0.058 ± 0.029 [149]	86 ± 4 [113]	150 ± 70 [115]	2.4 ± 2.4 [96]
MW 7000 [500 mg] *n* =6	0.27 ± 0.08 [-]	1.79 ± 0.13 [106]	0.04 ± 0.007 [103]	66 ± 5 [87]	143 ± 16 [110]	2.3 ± 0.6 [92]
**NIST SRM 1566a**						
**Certified** (*indicative/noncertified)	**0.2025 ± 0.0125**	**1.18 ± 0.017**	**4.15 ± 0.38**	**830 ± 57**	*400	*10
MARS 6 [100 mg] *n*=4	0.159 ± 0.009 [79]	1.30 ± 0.11 [110]	3.89 ± 0.25 [88]	879 ± 23 [106]	430 ± 70 [-]	13 ± 4 [-]
MW 7000 [100 mg] *n* =6	0.165 ± 0.016 [81]	0.91 ± 0.04 [77]	3.9 ± 0.3 [94]	930 ± 50 [112]	460 ± 40 [-]	16 ± 6 [-]
MW 7000 [500 mg] *n* =6	0.26 ± 0.03 [128]	1.35 ± 0.07 [114]	3.13 ± 0.15 [75]	686 ± 21 [77]	470 ± 60 [-]	14.4 ± 2.5 [-]
**NIST SRM 2976**						
**Certified** (*indicative/noncertified)	*0.134 ± 0.034	*5.3 ± 0.5	**0.82 ± 0.16**	**137 ± 13**	*109 ± 8	*2.4 ± 0.3
MARS 6 [100 mg] *n*=17	0.14 ± 0.04 [-]	5.0 ± 0.9 [-]	0.97 ± 0.20 [118]^#^	148 ± 29 [108]^#^	100 ± 19 [-]	2.0 ± 1.4 [-]
MW 7000 [100 mg] *n* =6	0.19 ± 0.16 [-]	3.98 ± 0.23 [-]	1.03 ± 0.14 [126]	155 ± 6 [113]	130 ± 50 [-]	2.3 ± 1.6 [-]
MW 7000 [500 mg] *n* =6	0.155 ± 0.020 [-]	4.24 ± 0.27 [-]	0.83 ± 0.06 [101]^#^	128 ± 8 [93]^#^	115 ± 13 [-]	2.4 ± 0.6 [-]

### Test portion sizes of 250 mg and 500 mg

Regarding the digestions with sample weights of 500 mg (NCS -ZC73034, NIST SRM 1566(a) and NIST SRM 2976) and 250 mg (BCR-668) using the Multiwave 7000 system (280 °C) recoveries are within the 70% - 125% range for all certified elements, except Al (128%) for NIST SRM 1566a (see Table 4). Relative uncertainties are in the range of 3% (Zn, NIST SRM 1566a) – 29% (Eu, BCR-668) with a median of 8%. For the certified elements Cd and Zn (NIST SRM 2976) and Ce and Eu (BCR-668) no significant difference from the certified value was found according to ERM [33]. Indicative/ noncertified mass fractions where uncertainty is available are within the uncertainty of mass fractions obtained in this study, except for the mass fraction of Mg in NIST SRM 2976: 4.24 g kg^-1^ ± 0.27 g kg^-1^ (this study) 5.3 g kg^-1^ ± 0.5 g kg^-1^ (indicative/ noncertified value).

### Test portion size of 100 mg

Regarding the digestions of sample weights of 100 mg using the MARS 6 system (200 °C), achieved recoveries are within 70% - 125% for all certified elements, except Eu for NCS ZC73034 (68%). Relative uncertainties are in the range of 3% (Zn, NIST SRM 1566a) – 57% (Eu, BCR-668) with a median of 21%. For the certified elements Cd and Zn (NIST SRM 2976) and Ce and Eu (BCR-668) no significant difference from the certified value was found according to ERM [33]. Indicative/ noncertified mass fractions where uncertainty is available are within the uncertainty of mass fractions obtained in this study for all elements discussed in Table 4.

The Multiwave 7000 system (280 °C) achieved recoveries within 70% - 125% for all certified elements, except Cd for NIST SRM 2976 (126%) and NCS ZC73034 (149%) (see Table 4). Relative uncertainties are in the range of 3% (Mg, NIST SRM 1566a) – 96% (EU, NCS ZC73034) with a median of 8%. For the certified elements Ce and Eu (BCR-668) no significant difference from the certified value was found according to ERM [33]. However, results for certified elements Cd and Zn (NIST SRM 2976) showed a significant difference to the certified values. Indicative/ noncertified mass fractions where uncertainty is available are within the uncertainty of mass fractions obtained in this study, except for the mass fraction of Mg in NIST SRM 2976: 3.98 g kg^-1^ ± 0.23 g kg^-1^ (this study) 5.3 g kg^-1^ ± 0.5 g kg^-1^ (indicative/ noncertified value).

### Optimum test portion size

Best results were obtained with sample weights of 500 mg (250 mg for BCR-668) using the Multiwave 7000 system (280 °C). Only one recovery was outside the 70% - 125% range and the lowest uncertainty on average was achieved. No significant differences between certified and measured mass fractions were found for elements eligible for evaluation [33]. Overall this method showed the best results, but also consumes a larger amount of sample material. Hence, we suggest this method for validation purposes or special application where it is necessary to ensure the best recovery with the lowest possible uncertainty and thus provide the most accurate mass fractions for TCEs. Results presented and recoveries discussed in the following chapters correspond to this method (for parameters see Table 2).

A lower sample weight of 100 mg in conjunction with the Multiwave 7000 system (280 °C) resulted in the poorest results, with two recoveries outside the 70% - 125% range and the highest uncertainty on average. Significant differences between measured and certified mass fractions were found for Cd and Zn (NIST SRM 2976) [33]. The higher uncertainties could be related to the lower sample masses, which were below the minimum test portion size given by the certificates for all reference materials except BCR-668.

The lower sample weight of 100 mg in conjunction with the MARS 6 system (200 °C) resulted in better results, with a total of only one recovery outside the 70% - 125% range and lower uncertainties on average compared to the method using the Multiwave 7000 and a 100 mg test portion size. No significant differences between measured and certified mass fractions were found [33].

Considering the lower amount of sample material needed, which confers an advantage when working with samples of limited portion size, while still obtaining high-quality results our laboratory uses the MARS 6 system for routine analysis. We consider this method superior for a daily routine allowing triplicate digestions, especially due to its effectiveness in cases with a total sample amount of only 300 mg. Obtained mass fractions using 100 mg sample weight can be found in the Supplementary Information 1 Tables ESM4 – ESM7.

### BCR-668 mussel tissue

Mass fractions of all analyzed relevant elements and corresponding recoveries can be found in Table 5. Recoveries of the certified REEs are within 90% - 110% for La, Ce, Pr, Sm, Eu, Gd, Tb, Dy and Er. Nd, Tm and Lu showed recoveries of 89%, 125% and 129%, respectively. Mean values show no significant difference from the certified values of REE [33]. Indicative mass fractions of Ho and Yb lay within the expanded uncertainty of the obtained mass fractions. Compared to the other analyzed reference materials BCR-668 showed slightly higher uncertainties for the heavy REEs, which could be due to the reduced sample weight (250 mg) in comparison to the other reference materials (500 mg). BCR-668 has no certified values for the less studied TCEs, therefore we suggest 11 µg kg^-1^ ± 9 µg kg^-1^ for Ga, 4.2 µg kg^-1^ ± 2.5 µg kg^-1^ for Ge, 5 µg kg^-1^ ± 4 µg kg^-1^ for Nb, 0.7 µg kg^-1^ ± 0.8 µg kg^-1^ for In and 0.5 µg kg^-1^ ± 1.0 µg kg^-1^ for Ta. Low mass fractions with a high uncertainty as given for In and Ta are typically an indication for values close to the LOQ. To the best of our knowledge, no mass fractions of TCEs outside the certificate have been published up to this point (July 2023).

**Table 5:** Certified (*indicative/noncertified) and measured mass fractions (dry mass) (*n*=6) of the analyzed reference materials BCR-668 (250 mg), NCS ZC73034 (500 mg), NIST SRM 1566a (500 mg) and NIST SRM 2976 (500 mg), processed by closed vessel digestion at 280 °C and 140 bar. Errors of the measured mass fractions correspond to the expanded uncertainty *U* (*k* = 2). # indicate measured mean values not significantly different from the certified value based on [33]

	BCR-668			NCS ZC73034			NIST SRM 1566a		NIST SRM 2976	
	Certified range / µg kg^-1^	Measured / µg kg^-1^	Recovery / %	Certified range / µg kg^-1^	Measured / µg kg^-1^	Recovery / %	Certified range / µg kg^-1^	Measured / µg kg^-1^	Certified range / µg kg^-1^	Measured / µg kg^-1^
Ga	-	11 ± 9	-	-	50 ± 17	-	-	67 ± 8	-	30 ± 7
Ge	-	4.2 ± 2.5	-	6 ± 1.4	5 ± 4	83	-	9 ± 4	-	31 ± 7
Nb	-	5 ± 4	-	16.5 ± 4	21 ± 4	127	-	39 ± 7	-	12.6 ± 1.9
In	-	0.7 ± 0.8	-	-	0.4 ±0.3	-	-	0.6 ± 0.5	-	0.8 ± 0.7
La	80 ± 6	77 ± 13	96^#^	66 ± 5	67 ± 8	102	*300 (0.3 µg g^-1^)	320 ± 30	-	63 ± 4
Ce	89 ± 7	92 ± 23	103^#^	130 ± 30	143 ± 16	110	*400 (0.4 µg g^-1^)	470 ± 60	*109 ± 8	115 ± 13
Pr	12.3 ± 1.1	13 ± 3	106^#^	14.5 ± 1.1	15.9 ± 1.9	110	-	74 ± 8	-	13.4 ± 1.2
Nd	54 ± 4	48 ± 9	89^#^	56 ± 6	52 ± 4	93	-	271 ± 25	-	50 ± 6
Sm	11.2 ± 0.8	11 ± 3	98^#^	10.7 ±1.8	10.1 ± 2.1	94	*60 (0.06 µg g^-1^)	63 ± 8	-	9.9 ± 2.3
Eu	2.79 ± 0.16	2.6 ± 0.8	93^#^	2.5 ± .0.3	2.3 ± 0.6	92	*10 (0.01 µg g^-1^)	14.4 ±2.5	*2.4 ± 0.3	2.4 ± 0.6
Gd	13 ± 0.6	12 ± 2.4	92^#^	10.5 ± 1.2	8 ± 3	76	-	63 ± 5	-	10.6 ± 2.2
Tb	1.62 ± 0.12	1.6 ± 0.5	99^#^	1.5 ± 0.2	1.2 ± 0.4	80	*7 (0.007 µg g^-1^)	10.1 ± 1.0	-	1.53 ± 0.28
Dy	8.9 ± 0.6	8.5 ± 2.2	96^#^	7.9 ± 0.5	7.6 ± 1.2	96	-	60 ± 5	-	8.1 ± 2.4
Ho	*1.8 ± 0.6	1.8 ± 0.6	-	1.5 ± 0.2	1.4 ± 0.3	93	-	12.3 ± 2.0	-	1.7 ± 0.4
Er	4.5 ± 0.5	4.8 ± 2.7	107^#^	4.4 ± 0.4	4.1 ± 1.4	93	-	38 ± 6	-	5.1 ± 1.2
Tm	0.48 ± 0.08	0.6 ± 0.4	125^#^	0.69 ± 0.18	0.6 ± 0.3	84	-	5.1 ± 0.9	-	0.64 ± 0.23
Yb	*2.8 ± 0.5	2.7 ± 2.1	-	4.1 ± 0.8	3.2 ± 1.3	78	-	30 ± 6	-	3.0 ± 1.6
Lu	0.389 ± 0.024	0.5 ± 0.5	129^#^	0.64 ± 0.21	0.5 ± 0.13	78	-	5 ± 0.9	-	0.46 ± 0.19
Ta	-	0.5 ± 1	-	-	1.0 ± 1.8	-	*3 (0.003 µg g^-1^)	0.8 ± 1.4	-	0.4 ± 0.8

### NCS ZC73034 prawn

This reference material has a broad variety of certified mass fractions for the analyzed TCEs including mass fractions for all REE, as well as for Ge and Nb. Mass fractions for all relevant elements and corresponding recoveries are shown in Table 5. Overall recoveries from 76% for Gd to 127% for Nb were obtained. Heavy REEs featured slightly lower recoveries compared to BCR-668 with Lu showing a recovery of 78%. Overall, the certified TCE mass fractions are within the uncertainty budget of this study. Significance between measured and certified mass fractions could not be assessed due to missing information in the certificate. For Ga, In, and Ta we suggest mass fractions of 50 µg kg^-1^ ± 17 µg kg^-1^, 0.4 µg kg^-1^ ± 0.3 µg kg^-1^ and 1.0 µg kg^-1^ ± 1.8 µg kg^-1^, respectively. Low mass fractions with a high uncertainty as given for In and Ta are normally an indication for values close to the LOQ. To the best of our knowledge no Ga, In, nor Ta mass fractions in NCS ZC73034 have been published up to the time of this study (July 2023).

### NIST SRM 1566a oyster tissue

NIST SRM 1566a has indicative mass fractions for the elements La, Ce, Sm, Eu, Tb and Ta. Measured mass fractions for all relevant elements can be found in Table 5. The indicative mass fraction of La is within the uncertainty budget. Measured mass fractions of Ce, Sm, Eu and Tb are slightly higher compared to the indicative values. The presented Ta mass fraction is lower than the indicative value. NIST SRM 1566a is the oldest reference material of this study and indicative values given in the certificate are not defined according to current guidelines. This reference material has previously been analyzed in other studies. Laborda *et al.* presented mass fractions of REEs as a result of their studies testing different carrier gas flows and additional nitrogen in the argon plasma in order to reduce polyatomic interferences in ICP-MS [34]. Additionally, Tormen *et al.* published a Ga mass fraction as a result of their studies of trace elements in biological samples treated with formic acid [35]. Indicative values, suggested values and literature values for NIST SRM 1566a can be found in Table 6. Our suggested Ga mass fraction (67 µg kg^-1^ ± 8 µg kg^-1^) is within the uncertainty of the literature value (60 µg kg^-1^ ± 14 µg kg^-1^). Literature values for REEs as published by Laborda *et al.* are slightly lower than the indicative values for La, Ce and Sm and within the uncertainty for Eu. Compared to the data of this study, higher precision was achieved for La, Ce and Sm in relation to the indicative values compared to Laborda *et al.. *The given literature value for Eu is slightly above the indicative value, within measurement uncertainty, indicating a possible overestimation of the Eu mass fraction for NIST SRM 1566a presented in this study. Overall, literature values for light REEs appear to be slightly lower than the results of this study, which can additionally be seen for the elements Pr and Nd. Pr is given with a significantly lower mass fraction in the literature, but within the same order of magnitude as the suggested value. In contrast, the mass fractions of heavy REEs from literature do not appear to follow the trend previously described for light REEs. Excluding Gd and Dy, mass fractions of heavy REEs are in good accordance with our suggested mass fractions and are within the expanded uncertainty. Mass fractions for Tb are above the indicative value both within this study, and within the study by Laborda *et al*.. The cross validation of the certified values for REE mass fractions of BCR-668 and NCS ZC73034 do not confirm an overestimation for Eu and Tb. The lower mass fraction of Ta compared to the indicative value is difficult to evaluate due to the lack of a given uncertainty. Furthermore, to the best of our knowledge no literature values of Ta in NIST SRM 1566a are available (July 2023). However, very similar mass fractions of Ta were found in all analyzed reference materials with relatively high uncertainty, indicating values near the LOQ. Without a given uncertainty for the indicative values, further comparison is not possible. Overall, the values presented here are determined with a high precision and are comparable to other studies.

**Table 6:** Comparison of certified (*indicative), suggested (*n*=6) and literature values for NIST SRM 1566a. Errors correspond to the expanded uncertainty *U* (*k* = 2), if not marked otherwise

	Certified range / µg kg^-1^	Measured / µg kg^-1^	Literature values / µg kg^-1^ ± standard deviation of duplicates [34] ± confidence interval of 95% [35]
Ga	-	67 ± 8	60 ± 14 [35]
Ge	-	9 ± 4	-
Nb	-	39 ± 7	-
In	-	0.6 ± 0.5	-
La	*300	320 ± 30	164 ± 5 – 234 ± 14 [34]
Ce	*400	470 ± 60	259 ± 5 – 312 ± 2 [34]
Pr	-	74 ± 8	45 ± 6 – 54 ± 2 [34]
Nd	-	271 ± 25	205 ± 36 – 236 ± 1 [34]
Sm	*60	63 ± 8	45 ± 3 – 52 ± 0 [34]
Eu	*10	14.4 ±2.5	11 ± 1 [34]
Gd	-	63 ± 5	52 ± 1 – 54 ± 0 [34]
Tb	*7	10.1 ± 1.0	9 ± 1 – 11 ± 0 [34]
Dy	-	60 ± 5	41 ± 3 – 53 ± 0 [34]
Ho	-	12.3 ± 2.0	11 ± 1 – 11 ± 2 [34]
Er	-	38 ± 6	33 ± 3 [34]
Tm	-	5.1 ± 0.9	5 ± 1 [34]
Yb	-	30 ± 6	29 ± 1 – 36 ± 13 [34]
Lu	-	5 ± 0.9	6 ± 2 [34]
Ta	*3	0.8 ± 1.4	-

### NIST SRM 2976 mussel tissue

Indicative mass fractions of Ce and Eu are given with the certificate for NIST SMR 2976. Both are in agreement with mass fractions presented in this study and within the uncertainty budget. Measured mass fractions for all relevant elements and corresponding recoveries can be found in Table 5.To the best of our knowledge, only one article was published up to this point (July 2023) suggesting mass fractions of TCEs in NIST SRM 2976. Krishna and Arunachalam [36] tested an ultrasound-assisted extraction sample preparation method prior to ICP-MS and ICP-AES (atomic emission spectrometry), and proposed a resulting Ge mass fraction of 68 µg kg^-1^ ± 5 µg kg^-1^ and an In mass fraction of 24 µg kg^-1^ ± 5 µg kg^-1^ , obtained via microwave digestion [36]. Both values are significantly higher than the ones we present in this study (Ge: 30 µg kg^-1^ ± 7 µg kg^-1^ and In: 0.8 µg kg^-1^ ± 0.7 µg kg^-1^. Indeed, Ge mass fractions of this study can be cross validated with NCS ZC73034 with a recovery of 83%. For In an evaluation is more challenging due to missing reference values. We found similar In mass fractions throughout all analyzed CRMs (0.4 µg kg^-1^ ± 0.3 µg kg^-1^ - 0.8 µg kg^-1^ ± 0.7 µg kg^-1^), indicating that a mass fraction of < 1 µg kg^-1^ may be plausible for marine organisms. Klein *et al.* published In mass fractions in sediment from the North Sea in the range of 73 µg kg^-1^ ± 4 µg kg^-1^ to 237 µg kg^-1^ ± 13 µg kg^-1^, the lower value being in the same order of magnitude as the In mass fraction in NIST SRM 2976 published by Krishna and Arunachalam [6, 36]. This might indicate that the In mass fraction published by Krishna and Arunachalam is overestimated, based on the fact that most elemental mass fractions in organisms tend to be orders of magnitude lower than in sediments, e.g.: Bell *et al.* published In mass fractions within *Corophium volutator* of 1 µg kg^-1^ – 5 µg kg^-1^ (<LOQ), which is in good accordance to the In mass fractions suggested within this study (0.4 µg kg^-1^ ± 0.3 µg kg^-1^ - 0.8 µg kg^-1^ ± 0.7 µg kg^-1^) [37]. As stated before and also seen in Bell *et al.* low mass fractions associated with high uncertainties indicate values near (or below) the LOQ, therefore further method development is needed. Overall, the lack of comparable data makes it difficult to ascertain which of the measured In mass fractions reflect the true value.

## Conclusion

As described previously, it is challenging to assess current levels and potential impacts of TCEs on the marine environment. Comparable data is scarce and few studies have been published in recent years related to the determination of TCEs, especially the less studied TCEs Ga, Ge, Nb, In, Te and Ta [23, 38]. The limited data availability presents a challenge when assessing the impact of metals on the marine environment and specifically on marine biota. Mussels in particular have a long history of use as bioindicators [39–41]. The determination of metal mass fractions in mussels is also found among first studies investigating possible environmental impacts of corrosion protection of offshore wind farms on marine biota [42, 43]. To support monitoring programs and the investigation of possible effects of increasing demand for TCEs, it is essential to ensure available reference values for CRMs. The digestion method performed in this study allows routine measurement of TCEs in marine organisms as part of a time and cost-efficient workflow in combination with ICP-MS/MS (in particular with N_2_O as a reaction cell gas). Commonly known interferences for REEs and TCEs are prevented, which allows existing CRMs to be applied to a broader selection of analytes, thereby improving the monitoring of TCEs in the marine environment. Furthermore, an additional digestion method is presented, allowing triplicate digestions with a total sample amount of only 300 mg, which confers an advantage when working with samples of limited portion size, while still obtaining high-quality results with slightly higher uncertainties. Even though NCS ZC73034 (as the newest CRM analyzed in this study) possesses a large number of certified elemental mass fractions, important TCEs such as Ga, In and Ta are not among the certified elements. To the best of our knowledge (July 2023) no other reference materials in the field of marine environment delivers certified mass fractions for these three elements (Ga, In and Ta) (NIST SRM 1566a offers only a noncertified value and is no longer commercially available). Therefore, the production of new reference materials or the re-certification of existing ones for mass fractions of TCEs remains of high interest to the scientific community.

### Supplementary Information

Below is the link to the electronic supplementary material.

Supplementary file1 (DOCX 71 KB)

Supplementary file2 (XLSX 28 KB)
