# Supplementary material for: Determination of technology-critical elements in seafood reference materials by inductively coupled plasma-tandem mass spectrometry
Source: Anal Bioanal Chem. 2023 Dec 23;416(11):2797–807. doi: 10.1007/s00216-023-05081-z (PMC11009730; doi:10.1007/s00216-023-05081-z)
Supplement: Supplementary file 1 — Supplementary file1 (DOCX 71 KB) [file 216_2023_5081_MOESM1_ESM.docx]

**Supplementary Information**

**Determination of technology-critical elements in seafood reference materials by inductively coupled plasma-tandem mass spectrometry**

Dominik Wippermann, Alexa Zonderman, Tristan Zimmermann,
 Daniel Pröfrock^*^

* Corresponding author: daniel.proefrock@hereon.de

**ORCID**

Dominik Wippermann: 0009-0008-0960-0543

Alexa Zonderman: 0009-0004-9080-5212

Tristan Zimmermann: 0000-0002-2302-7519

Daniel Pröfrock: 0000-0003-2741-3027

**Table ESM 1:** Overview of available seafood CRMs. Literature is only presented if mass fractions of elements from this study are given (printed in bold). Indicative/noncertified mass fractions and additional material information are written italic.

| CRM | Certified analytes (including indicative values) | Reference | Method | Analyzed elements |
| --- | --- | --- | --- | --- |
| Crustaceans and mollusks | | | | |
| BCR-668  (Mussel tissue) | *As*, *Cd*, **Ce,** *Co*, *Cr*, *Cs*, **Dy,** **Er, Eu,** *Fe*, **Gd, *Ho*, La, Lu,** *Mo*, **Nd,** **Pr,** *Sc*, **Sm,** **Tb,** Th, **Tm,** U, ***Yb*,** *Zn* | **-** | **-** | **-** |
| ERM-CE278K (Mussel tissue) | As, *Ag*, *Ca*, Cd, *Cl*, *Co*, Cr, Cu, Fe, Hg, *K*, *Mg*, Mn, *Na*, Ni, Pb, Rb, Se, Sr, Zn | **-** | **-** | **-** |
| IAEA-461 (Clam) | *Al*, As, Ca, Cd, Co, Cr, Cu, Fe, Hg, *Mg*, Mn, Ni, Pb, Se, *Sr*, V, Zn | **-** | **-** | **-** |
| NCS ZC73034  (Prawn) | *Ag*, *Al*, *As*, B, Ba, Be, *Bi*, Br, Ca, Cd, **Ce,** *Cl*, Co, Cr, Cs, Cu, **Dy, Er, Eu**, Fe, **Gd, Ge,** Hg, **Ho,** *I*, K, **La,** Li, **Lu,** Mg, Mn, Mo, *N*, Na, **Nb, Nd,** *Ni*, P, Pb, **Pr,** Rb, *S*, *Sb*, *Sc*, *Se*, *Si*, **Sm,** *Sn*, Sr, **Tb,** Th, *Ti*, Tl, **Tm,** U, V, Y, **Yb,** Zn | **-** | **-** | **-** |
| NIST SRM 1566a  (Oyster tissue) | Ag, Al, As, *Au*, Ca, Cd, ***Ce*,** Co, Cr, *Cs*, Cu, ***Eu*,** *F*, Fe, *Hf*, Hg, I, K, ***La*,** Mg, Mn, *N*, Na, Ni, P, Pb, *Rb*, S, *Sb*, *Sc*, Se, ***Sm*,** *Sn*, Sr, ***Ta*,** ***Tb*,** *Th*, U, V, Zn | Laborda *et al.* (1994) [23] | Microwave assisted sample digestion | **Ce,** **Dy, Eu, Er,** **Gd,** **Ho,** **La,** **Lu,** **Nd,** **Pr,** **Sm,** **Tb,** **Tm,** **Yb** |
|  |  | Tormen *et al.* (2010) [24] | Microwave assisted sample digestion | **Ga** |
| NIST SRM 2976  (Mussel tissue) | *Ag*, *Al*, As, *Br*, *Ca*, Cd, ***Ce*,** *Cl*, *Co*, *Cr*, *Cs*, Cu, ***Eu*,** Fe, Hg, *K*, *Mg*, *Mn*, *Na*, *Ni*, *P*, Pb, *Rb*, *S*, *Sc* Se, *Sn*, *Sr*, *Th*, *Tl*, Zn | Krishna and Arunachalam (2004) [25] | Ultrasonic probe and microwave digestion | **Ge, In** |
| Fish | | | | |
| ERM-BB422 (Fish muscle) | As, *Ca*, *Cl*, Cd, Cu, Fe, Hg, I, *Mg*, Mn, *Na*, *K*, Se, Zn | **-** | **-** | **-** |
| NRC-DOLT-5 (Dogfish liver) | Ag, *Al*, As, Ca, Cd, Co, *Cr*, Cu, Fe, *Hg*, K, Mg, *Mn*, Mo, Na, *Ni*, *P*, Pb, *Sb*, Se, Sn, Sr, *Tl*, *U*, V, Zn | **-** | **-** | **-** |
| NRC-DORM-5 (Fish protein) | Ag, Al, As, Ba, B, *Br*, Ca, Cd, *Cl*, Co, Cr, Cu, *I*, Fe, K, Li, Mg, Mn, Na, Ni, P, Pb, Rb, *S*, *Sb*, Se, Sn, Sr, U, V, Zn | **-** | **-** | **-** |

**Table ESM 2:** Instrument settings and operating conditions of the Agilent 8800 ICP-MS/MS.

| **Instrument parameters** | **Operating settings and conditions** |
| --- | --- |
| Sample introduction | Double-pass spray chamber |
| Nebulizer | Self-aspirating MicroFlow (ESI) |
| Interface cones | Nickel |
| RF power (W) | 1550 |
| Carrier gas flow (L min^-1^) | 1.07 |
| Make-up gas flow (L min^-1^) | 0.1 |
| Used cell gases | He_,_ N_2_O |
| Extract 1 (V) | 0 |
| Extract 2 (V) | -175 |
| Omega Bias (V) | -100 |
| Omega Lens (V) | 9.6 |
| Q1 Entrance (V) | H_2_: 1 He: -1; N_2_O: 1 |
| Q1 Exit (V) | H_2_: -2 He: -9; N_2_O: -2 |
| Cell Focus (V) | H_2_: 1 He: 2; N_2_O: 7 |
| Cell Entrance (V) | H_2_: -50 He: -50; N_2_O: -40 |
| Cell Exit (V) | H_2_: -60 He: -60; N_2_O: -51 |
| Deflect (V) | H_2_: -3.4 He: -4.8; N_2_O: 8.8 |
| Plate Bias (V) | H_2_: -60 He: -60; N_2_O: -50 |
| Cell gas flow (mL min^-1^) | H_2_: 6.0 He: 4.5; N_2_O: 20% |
| Octpole Bias (V) | H_2_: -18 He: -20; ; N_2_O: 0.5 |
| Octpole RF (V) | H_2_: 170 He: 200; N_2_O: 170 |
| Energy Discrimination (V) | H_2_: 0 He: 5; N_2_O: -5 |

**Table ESM 3:** ICP-MS/MS measured mass-to-charge ratios and gas modes adopted for multi-elemental analysis in this study.

| **Element** | ***m/z* (Q1)** | ***m*/*z* (Q2)** | **cell mode** |
| --- | --- | --- | --- |
| Mg | 24 | 24 | H_2_ |
| Al | 27 | 27 | H_2_ |
| Zn | 66 | 66 | He |
| Ga | 71 | 71 | He |
| Ge | 72 | 88 | N_2_O |
| Nb | 93 | 125 | N_2_O |
| Cd | 111 | 111 | He |
| In | 115 | 115 | N_2_O |
| La | 139 | 155 | N_2_O |
| Ce | 140 | 156 | N_2_O |
| Pr | 141 | 157 | N_2_O |
| Nd | 146 | 162 | N_2_O |
| Sm | 147 | 163 | N_2_O |
| Eu | 153 | 169 | N_2_O |
| Gd | 157 | 173 | N_2_O |
| Tb | 159 | 175 | N_2_O |
| Dy | 163 | 179 | N_2_O |
| Ho | 165 | 181 | N_2_O |
| Er | 166 | 182 | N_2_O |
| Tm | 169 | 185 | N_2_O |
| Yb | 172 | 188 | N_2_O |
| Lu | 175 | 191 | N_2_O |
| Ta | 181 | 213 | N_2_O |

**Table ESM 4:** Overview of analyzed mass fractions (dry mass) of BCR-668 for rare earth elements/TCEs for all tested method parameters.

| BCR-668 | | | | | | | | | | |  |
| --- | --- | --- | --- | --- | --- | --- | --- | --- | --- | --- | --- |
| Element | Certified (*indicative) range / µg/kg | MW7000 (250 mg) *n*=6 | | | MW7000 (100 mg) *n*=6 | | | Mars 6 (100 mg) *n*=16 | | |  |
|  |  | Measured / µg/kg | Uncertainty *U* (*k*=2) / µg/kg | Recovery / % | Measured / µg/kg | Uncertainty *U* (*k*=2) / µg/kg | Recovery / % | Measured / µg/kg | Uncertainty *U* (*k*=2) / µg/kg | Recovery / % |  |
|  |  |  |  |  |  |  |  |  |  |  |  |
| Ga | - | 11 | 9 | - | 30 | 40 | - | 12 | 13 | - |  |
| Ge | - | 4.2 | 2.5 | - | - | - | - | - | - | - |  |
| Nb | - | 5 | 4 | - | - | - | - | 1.4 | 1.1 | - |  |
| In | - | 0.7 | 0.8 | - | 0.5 | 2.2 | - | 1.1 | 1.6 | - |  |
| La | 80 ± 6 | 77 | 13 | 96 | 87 | 24 | 109 | 77 | 22 | 96 |  |
| Ce | 89 ± 7 | 92 | 23 | 103 | 110 | 50 | 124 | 87 | 21 | 98 |  |
| Pr | 12.3 ± 1.1 | 13 | 3 | 106 | 15 | 7 | 122 | 14 | 6 | 114 |  |
| Nd | 54 ± 4 | 48 | 9 | 89 | 55 | 25 | 102 | 48 | 17 | 89 |  |
| Sm | 11.2 ± 0.8 | 11 | 3 | 98 | 11 | 6 | 98 | 11 | 7 | 98 |  |
| Eu | 2.79 ± 0.16 | 2.6 | 0.8 | 93 | 2.6 | 1.8 | 93 | 2.6 | 1.6 | 93 |  |
| Gd | 13 ± 0.6 | 12 | 2.4 | 92 | 13 | 8 | 100 | 12 | 7 | 92 |  |
| Tb | 1.62 ± 0.12 | 1.6 | 0.5 | 99 | 1.8 | 1.2 | 111 | 1.5 | 1.3 | 93 |  |
| Dy | 8.9 ± 0.6 | 8.5 | 2.2 | 96 | 10 | 9 | 112 | 8 | 6 | 90 |  |
| Ho | *1.8 ± 0.6 | 1.8 | 0.6 | - | 1.8 | 1.5 | - | 1.6 | 1.2 | - |  |
| Er | 4.5 ± 0.5 | 4.8 | 2.7 | 107 | 7 | 7 | 156 | 4 | 3 | 89 |  |
| Tm | 0.48 ± 0.08 | 0.6 | 0.4 | 125 | 0.8 | 1.1 | 167 | 0.5 | 0.6 | 104 |  |
| Yb | *2.8 ± 0.5 | 2.7 | 2.1 | - | 5 | 7 | - | 2 | 4 | - |  |
| Lu | 0.389 ± 0.024 | 0.5 | 0.5 | 129 | 1.1 | 1.3 | 283 | 0.4 | 0.5 | 103 |  |
| Ta | - | 0.5 | 1 | - | - | - | - | - | - | - |  |

**Table ESM 5:** Overview of analyzed mass fractions (dry mass) of NCS ZC73034for rare earth elements/TCEs for all tested method parameters.

| NCS ZC73034 | | | | | | | | | | |  |
| --- | --- | --- | --- | --- | --- | --- | --- | --- | --- | --- | --- |
| Element | Certified  (*indicative) range / µg/kg | MW7000 (500 mg) *n*=6 | | | MW7000 (100 mg) *n*=6 | | | Mars 6 (100 mg) *n*=7 | | |  |
|  |  | Measured / µg/kg | Uncertainty *U* (*k*=2) / µg/kg | Recovery / % | Measured / µg/kg | Uncertainty *U* (*k*=2) / µg/kg | Recovery / % | Measured / µg/kg | Uncertainty *U* (*k*=2) / µg/kg | Recovery / % |  |
|  |  |  |  |  |  |  |  |  |  |  |  |
| Ga | - | 50 | 17 | - | 70 | 40 | - | 47 | 28 | - |  |
| Ge | 6 ± 1.4 | 5 | 4 | 83 | - | - | - | 2 | 8 | 33 |  |
| Nb | 16.5 ± 4 | 21 | 4 | 127 | - | - | - | 5.9 | 2.9 | 36 |  |
| In | - | 0.4 | 0.3 | - | - | - | - | 0.3 | 0.7 | - |  |
| La | 66 ± 5 | 67 | 8 | 102 | 70 | 30 | 106 | 59 | 8 | 89 |  |
| Ce | 130 ± 30 | 143 | 16 | 110 | 150 | 70 | 115 | 126 | 15 | 97 |  |
| Pr | 14.5 ± 1.1 | 15.9 | 1.9 | 110 | 18 | 8 | 124 | 15 | 4 | 103 |  |
| Nd | 56 ± 6 | 52 | 4 | 93 | 55 | 23 | 98 | 48 | 13 | 86 |  |
| Sm | 10.7 ±1.8 | 10.1 | 2.1 | 94 | 10 | 4 | 93 | 9 | 6 | 84 |  |
| Eu | 2.5 ± .0.3 | 2.3 | 0.6 | 92 | 2.4 | 2.4 | 96 | 1.7 | 1.2 | 68 |  |
| Gd | 10.5 ± 1.2 | 8 | 3 | 76 | 9 | 4 | 86 | 7 | 5 | 67 |  |
| Tb | 1.5 ± 0.2 | 1.2 | 0.4 | 80 | 1.5 | 1.3 | 100 | 0.9 | 0.5 | 60 |  |
| Dy | 7.9 ± 0.5 | 7.6 | 1.2 | 96 | 10 | 9 | 127 | 5.1 | 2.4 | 65 |  |
| Ho | 1.5 ± 0.2 | 1.4 | 0.3 | 93 | 2.3 | 2.2 | 153 | 1.2 | 0.9 | 80 |  |
| Er | 4.4 ± 0.4 | 4.1 | 1.4 | 93 | 6 | 7 | 136 | 3.2 | 2.1 | 73 |  |
| Tm | 0.69 ± 0.18 | 0.58 | 0.3 | 84 | 1 | 1.8 | 145 | 0.4 | 0.4 | 58 |  |
| Yb | 4.1 ± 0.8 | 3.2 | 1.3 | 78 | 5 | 8 | 122 | 2 | 3 | 49 |  |
| Lu | 0.64 ± .21 | 0.5 | 0.13 | 78 | 1.2 | 1.8 | 188 | 0.5 | 0.4 | 78 |  |
| Ta | - | 1 | 1.8 | - | - | - | - | - | - | - |  |

**Table ESM 6:** Overview of analyzed mass fractions (dry mass) of NIST SRM 1566a for rare earth elements/TCEs for all tested method parameters.

| NIST SRM 1566a | | | | | | | | | | |  |
| --- | --- | --- | --- | --- | --- | --- | --- | --- | --- | --- | --- |
| Element | Certified  (*indicative) range / µg/kg | MW7000 (500 mg) *n*=6 | | | MW7000 (100 mg) *n*=6 | | | Mars 6 (100 mg) *n*=4 | | |  |
|  |  | Measured / µg/kg | Uncertainty *U* (*k*=2) / µg/kg | Recovery / % | Measured / µg/kg | Uncertainty *U* (*k*=2) / µg/kg | Recovery / % | Measured / µg/kg | Uncertainty *U* (*k*=2) / µg/kg | Recovery / % |  |
|  |  |  |  |  |  |  |  |  |  |  |  |
| Ga | - | 67 | 8 | - | 100 | 70 | - | 71 | 15 | - |  |
| Ge | - | 9 | 4 | - | - | - | - | 4 | 10 | - |  |
| Nb | - | 39 | 7 | - | - | - | - | 11 | 4 | - |  |
| In | - | 0.6 | 0.5 | - | - | - | - | 3.1 | 1.4 | - |  |
| La | *300  (0.3 µg g^-1^) | 320 | 30 | - | 300 | 30 | - | 290 | 30 | - |  |
| Ce | *400  (0.4 µg g^-1^) | 470 | 60 | - | 460 | 40 | - | 430 | 70 | - |  |
| Pr | - | 74 | 8 | - | 77 | 6 | - | 68 | 11 | - |  |
| Nd | - | 271 | 25 | - | 270 | 17 | - | 260 | 40 | - |  |
| Sm | *60  (0.06 µg g^-1^) | 63 | 8 | - | 59 | 11 | - | 59 | 15 | - |  |
| Eu | *10  (0.01 µg g^-1^) | 14.4 | 2.5 | - | 16 | 6 | - | 13 | 4 | - |  |
| Gd | - | 63 | 5 | - | 80 | 60 | - | 65 | 13 | - |  |
| Tb | *7  (0.007 µg g^-1^) | 10.1 | 1 | - | 12 | 9 | - | 9.3 | 1.6 | - |  |
| Dy | - | 60 | 5 | - | 60 | 30 | - | 52 | 10 | - |  |
| Ho | - | 12.3 | 2 | - | 13 | 5 | - | 10.9 | 1.9 | - |  |
| Er | - | 38 | 6 | - | 34 | 9 | - | 29 | 7 | - |  |
| Tm | - | 5.1 | 0.9 | - | 5 | 3 | - | 4.1 | 1.2 | - |  |
| Yb | - | 30 | 6 | - | 32 | 15 | - | 24 | 10 | - |  |
| Lu | - | 5 | 0.9 | - | 5 | 2.2 | - | 4.3 | 1.3 | - |  |
| Ta | *3  (0.003 µg g^-1^) | 0.8 | 1.4 | - | - | - | - | - | - | - |  |

**Table ESM 7:** Overview of analyzed mass fractions (dry mass) of NIST SRM 2976 for rare earth elements/TCEs for all tested method parameters.

| NIST SRM 2976 | | | | | | | | | | |  |
| --- | --- | --- | --- | --- | --- | --- | --- | --- | --- | --- | --- |
| Element | Certified  (*indicative) range / µg/kg | MW7000 (500 mg) *n*=6 | | | MW7000 (100 mg) *n*=6 | | | Mars 6 (100 mg) *n*=17 | | |  |
|  |  | Measured / µg/kg | Uncertainty *U* (*k*=2) / µg/kg | Recovery / % | Measured / µg/kg | Uncertainty *U* (*k*=2) / µg/kg | Recovery / % | Measured / µg/kg | Uncertainty *U* (*k*=2) / µg/kg | Recovery / % |  |
|  |  |  |  |  |  |  |  |  |  |  |  |
| Ga | - | 30 | 7 | - | 50 | 40 | - | 34 | 17 | - |  |
| Ge | - | 31 | 7 | - | - | - | - | 31 | 20 | - |  |
| Nb | - | 12.6 | 1.9 | - | - | - | - | 4 | 3 | - |  |
| In | - | 0.8 | 0.7 | - | - | - | - | - | - | - |  |
| La | - | 63 | 4 | - | 67 | 24 | - | 59 | 14 | - |  |
| Ce | *109 ± 8 | 115 | 13 | - | 130 | 50 | - | 100 | 19 | - |  |
| Pr | - | 13.4 | 1.2 | - | 15 | 6 | - | 14 | 7 | - |  |
| Nd | - | 50 | 6 | - | 51 | 19 | - | 44 | 11 | - |  |
| Sm | - | 9.9 | 2.3 | - | 9 | 7 | - | 11 | 5 | - |  |
| Eu | *2.4 ± 0.3 | 2.4 | 0.6 | - | 2.3 | 1.6 | - | 2 | 1.4 | - |  |
| Gd | - | 10.6 | 2.2 | - | 10 | 7 | - | 10 | 6 | - |  |
| Tb | - | 1.53 | 0.28 | - | 1.4 | 1.3 | - | 1.4 | 1 | - |  |
| Dy | - | 8.1 | 2.4 | - | 8 | 6 | - | 8 | 4 | - |  |
| Ho | - | 1.7 | 0.4 | - | 2.1 | 1.6 | - | 1.6 | 1.2 | - |  |
| Er | - | 5.1 | 1.2 | - | 5 | 5 | - | 4 | 4 | - |  |
| Tm | - | 0.64 | 0.23 | - | 0.9 | 1.1 | - | 0.5 | 0.7 | - |  |
| Yb | - | 3 | 1.6 | - | 6 | 8 | - | 2 | 4 | - |  |
| Lu | - | 0.46 | 0.19 | - | 0.8 | 1.2 | - | 0.4 | 0.5 | - |  |
| Ta | - | 0.4 | 0.8 | - | - | - | - | - | - | - |  |
